# Influence of Magnet Particle Shape on Magnetic and Environmental Stability of FDM Polymer-Bonded Magnets

**DOI:** 10.3390/ma16082993

**Published:** 2023-04-10

**Authors:** Ana Damnjanović, Nataša Kovačević

**Affiliations:** 1 Jožef Stefan International Postgraduate School, SI-1000 Ljubljana, Slovenia; 2 Kolektor Mobility d.o.o., SI-5280 Idrija, Slovenia

**Keywords:** Nd–Fe–B, PA12-bonded magnets, additive manufacturing, fused deposition modelling, permanent magnets

## Abstract

In this research, the feasibility of additive manufacturing of permanent bonded magnets using fused deposition modelling (FDM) technology was investigated. The study employed polyamide 12 (PA12) as the polymer matrix and melt-spun and gas-atomized Nd–Fe–B powders as magnetic fillers. The effect of the magnetic particle shape and the filler fraction on the magnetic properties and environmental stability of polymer-bonded magnets (PBMs) was investigated. It was found that filaments for FDM made with gas-atomized magnetic particles were easier to print due to their superior flowability. As a result, the printed samples exhibited higher density and lower porosity when compared to those made with melt-spun powders. Magnets with gas-atomized powders and a filler loading of 93 wt.% showed a remanence (Br) of 426 mT, coercivity (Hci) of 721 kA/m, and energy product (BHmax) of 29 kJ/m^3^, while melt-spun magnets with the same filler loading had a remanence of 456 mT, coercivity of 713 kA/m, and energy product of 35 kJ/m^3^. The study further demonstrated the exceptional corrosion resistance and thermal stability of FDM-printed magnets, with less than 5% irreversible flux loss when exposed to hot water or air at 85 °C for over 1000 h. These findings highlight the potential of FDM printing for producing high-performance magnets and the versatility of this manufacturing method for various applications.

## 1. Introduction

Permanent magnets based on rare-earth materials are an indispensable component of modern technology. Not only do they provide higher-energy products than alnico or ferrite magnets, but they are also able to achieve this with less volume. They are the core of green technologies, such as hybrid and electric vehicles and wind turbines. Permanent magnets are most commonly manufactured using formative technologies, where magnetic powders can be compacted by sintering. Sintered magnets are known for their high-energy products but are prone to corrosion. Polymer-bonded magnets (PBMs) can be formed by applying pressure and high temperatures to the feedstock material by compression moulding, extrusion, injection moulding, or calendering processes. The feedstock material consists of a magnetic powder and a polymer binder. PBMs have some advantages over sintered magnets, as they can be easily fabricated into a near-net shape and have good mechanical properties. However, owing to the presence of the polymer, the magnetic properties of PBMs are lower compared to sintered magnets. A decrease in the remanence and energy of the product is linked to the amount of polymer used, as well as to the presence of pores and internal magnetic shear loss [1].

The major advantage of compression moulding is that it allows for a high magnetic filler loading. In the case of epoxy-bonded Nd–Fe–B magnets, the loading of the magnetic filler can be up to 98.5 wt.%. However, a disadvantage of compression moulding is that only simple geometries can be produced. On the other hand, injection moulding can produce complex shapes, although it requires specific tooling, which is costly [2]. Additive manufacturing (AM) technologies are promising solutions for overcoming the problem of expensive tooling. AM is a technology in which the desired shape is achieved by the successive addition of feedstock material, usually in a layer-by-layer manner [3]. With AM technology, there is great freedom in producing complex geometries, which enables the production of lighter parts. In the automotive industry, this feature is extremely important from both the environmental and economic perspectives. With lighter vehicles, fuel consumption and carbon emissions are reduced. Another economic aspect is that in additive technologies, material is built up and not removed, resulting in reduced material consumption. This is particularly significant for critical raw materials, such as magnetic powders based on rare-earth elements.

Material extrusion is by far the most commonly used AM technology. Common names include fused deposition modelling (FDM) and fused filament fabrication (FFF). In FDM, the feedstock material is in the form of a thermoplastic filament. The filament is loaded into the extruder, where it is heated, melted, and deposited through the nozzle head on the printer bed. Initially, filaments were made from various thermoplastic polymers. For instance, studies have been conducted on optimizing printing parameters to achieve tailored mechanical properties of composites based on polylactic acid and thermoplastic polyurethane [4,5]. Furthermore, the utilization of statistical evaluations to optimize process parameters for thermoplastic polymers can lead to achieving the best objective function [6]. Adopting a statistical approach in this regard can offer a time-saving advantage compared to experimental approaches. In recent years, FDM objects have been successfully printed using filaments made from composites, metals and alloys, ceramics, concrete, and biomaterials [7]. Magnetic fillers in PBMs can be bonded with various polymers depending on the final application. To date, FDM magnets have been printed in which an Nd–Fe–B powder is bonded with polyamide 11 [8], polyamide 12 [9], polyphenylene sulfide [10], ethylene ethyl acrylate [11], thermoplastic polyurethane [12], and polyether ether ketone [13]. The filament was also manufactured from a multicomponent system of polyoxymethylene as a dominant binder, mixed with spherical Nd–Fe–B powder and compounded Nd–Fe–B/polyamide 12 pellets [14]. Beside filaments, PBM can be extruded from slurries made of thermosetting epoxy resin [15] or photopolymer resin [16]. In this case, the FDM printing is coupled with UV curing. The manner in which the filaments are fused layer by layer causes most FDM objects to be anisotropic and not fully dense. Typically, there is a weaker bond and strength along the plane of the layer interface on a printed object [17].

The manufacturing of filaments is one of the greatest challenges in FDM. The filament for FDM needs to be extruded within a certain diameter and ovality tolerance to be printable at a constant flow rate over time. The filament needs to be stiff yet flexible enough to allow it to be spooled during filament production and despooled during printing. Finally, it must be homogenous. A complex filament production process can be avoided in big-area additive manufacturing (BAAM). BAAM is a material extrusion-based AM technology that uses pre-compounded materials in the form of pellets. BAAM is intended for printing very large objects, has a lower resolution, and it is expensive. BAAM-printed Nd–Fe–B/polyamide 12 magnets exhibit better magnetic properties than injection-moulded magnets [9].

An important characteristic of PBMs, in addition to having a high remanence and energy product, is their long-term stability and their ability to resist demagnetization, which consequently affects magnet performance during operation. Exposure to high temperatures or environmental degradation by corrosion leads to demagnetization. It is certainly important to consider corrosion resistance when selecting PBMs for use in harsh environments, especially in the automotive industry, where components are subjected to a wide range of conditions.

In addition, there is an interference adhesion problem. Conventional methods to improve adhesion in polymer-bonded magnets include the surface treatment of the magnetic powder. For instance, passivation pre-treatment, such as phosphatizing [18] or chromatizating [19], followed by coating magnetic powders in an aqueous solution of silanes [18,19,20,21]. These processes are performed via wet chemistry routes, which can be time-consuming and challenging to apply on a production scale. In this study, to improve the adhesion between the magnetic powder and the polymer binder, we decided to use all components in the same physical state.

We have investigated the influence of the magnetic particles shape as well as the magnetic filer fraction on the magnetic and mechanical properties and environmental stability of PBMs. With an aim to improve adhesion between magnetic filler particles and polymer binder, all feedstock material was applied in the powder form. Furthermore, the comparison of alternative additive manufacturing with the traditional production injection moulding technique was performed.

## 2. Materials and Methods

### 2.1. Filament Extrusion of Nd–Fe–B Bonded with Polyamide 12

Two commercial isotropic magnetic powders based on Nd–Fe–Co–B alloys were used for the FDM printing of the specimens. Both powders were provided by Magnequench. Mqp B+ powder (−150 mesh), based on a Nd–Fe–Co–B alloy, was produced using a melt-spinning process, generating particles with irregular flake-like morphology. Mqp B+ powder has a particle-size distribution predominantly below 90 microns, denoted by the negative sign before “150 mesh”, indicating that the powder particles were smaller than the mesh size. The Mqp S powder, based on a Nd–Pr–Fe–Co–Ti–Zr–B alloy, was produced by gas atomization; thus, the particles had a spherical morphology. The Mqp S powder has a d50 value in the range of 30–55 microns, signifying that half of the particles were larger than this range while the remaining half were smaller. Mqp S, being less coarse, is suitable for the manufacture of bonded magnets, particularly by injection moulding, extrusion, and calendering, owing to its superior flowability [22]. The properties of the Nd–Fe–B powders are summarized in Table 1. Mqp B+ has higher initial magnetic properties, which makes it more attractive for certain applications despite its potentially lower flowability compared to Mqp S. Polyamide 12 (PA12), a polymer binder, was used in the powder form (Vestosint, Evonik, Pandino, Italy). Magnetic and polymer powders were used as received without preconditioning, in other words, without drying, sieving, magnetizing, etc.

The amount of Nd–Fe–B powder in the bonded magnet is directly responsible for its magnetic and mechanical behaviours of bonded magnets. However, a higher content of magnetic filler may change the rheology of the polymer melt during the process and subsequently affect the mechanical properties of the bonded magnets. That is why this study utilized two different polymer loadings of 7 and 10 wt.%.

Because of the poor adhesion between the inorganic filler and the organic polymer matrix, titanium triisostearoylisopropoxide (TTS, Ken-React^®^ CAPOW^®^ KR^®^ TTS/H) was added to the filler as a powdered coupling agent. The role of TTS is to provide a molecular bridge at the interface between two substrates [23]. The magnetic filler was premixed in a kitchen blender with 1 wt.% TTS. After one minute of mixing, 0.2 wt.% zinc stearate (Sigma-Aldrich, St. Louis, MO, USA) and 0.5 wt.% stearic acid (Sigma-Aldrich) were added and mixed for one minute. Because the former acts as an external lubricant, whereas the latter serves as an internal lubricant, they were added to reduce the powder surface friction and to protect the extruder parts [24]. The final step involved blending a suitable amount of PA12 with a pre-prepared premix of magnetic powder and additives. The mixture was homogenized for a duration of two minutes.

**Figure 1:**
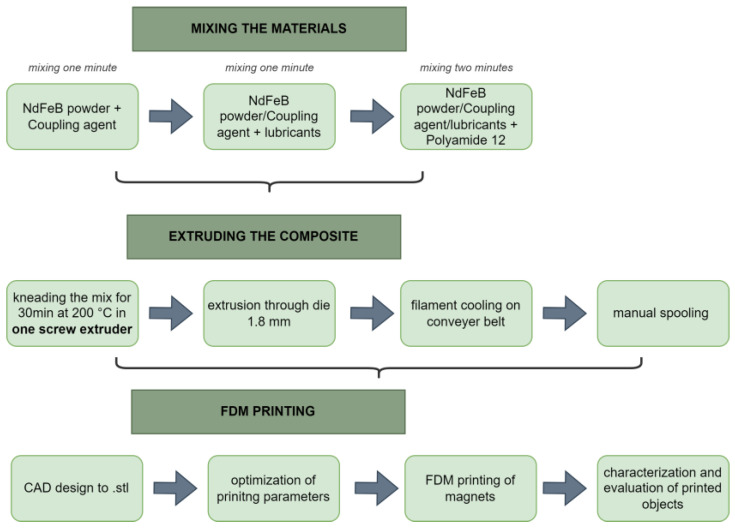
Schematic representation of the production processes for filaments and FDM printing.

Filament extrusion was achieved using a single-screw extruder (Linden IIKA). Inside the extruder vessel, there were double Z blades for kneading and homogenizing the material. The extruder was purged with a Dyna-Purge^®^ E2 cleaning mass between batches to prevent contamination of the subsequent batch. The premixed filler, additives, and polymer were flood-fed into the vessel and kneaded for 30 min at temperatures between 185 °C and 200 °C. The feedstock material was mixed, melted, and extruded through a die with a diameter of 1.8 mm. The filament was cooled using compressed air at the conveyor belt. The diameter of the filament was manually measured with a calliper at the end of the conveyer belt. It was kept in range between 1.7 and 1.8 mm by adjusting the speed of the conveyer belt. Table 2 provides a summary of four batches that were extruded into printable filaments, while Figure 1 depicts a schematic representation of the manufacturing process involved in the production of filaments and FDM printing.

### 2.2. Additive Manufacturing of Permanent Magnets

**Figure 2:**
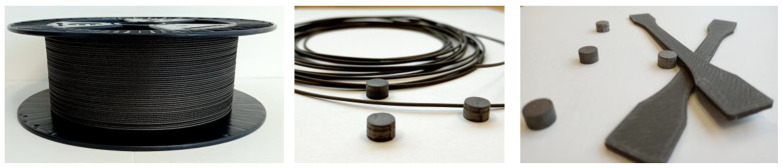
Extruded filament for FDM and FDM-printed samples in the form of cylinders and dog-bone-shaped test tubes.

In FDM printing, nozzle clogging can lead to interruptions in the printing process, rendering it impossible to clean the nozzle and resume printing on the same sample. This issue is particularly pronounced when dealing with highly filled filaments, such as those utilized in our study. As a result, our foremost goal was to ensure uninterrupted printing of each sample. Therefore, the determination of printing parameters was driven by the need to sustain continuous FDM printing while avoiding any nozzle clogging complications. For the FDM extrusion of the bonded magnets, a commercial desktop Craftbot Flow XL 3D printer was used. The filaments were extruded at 250 °C and the printing speed was 10 mm/s. The printing platform was heated to 70 °C to ensure additional adhesion of the extruded material. To avoid clogging during material extrusion, a nozzle head with a 0.8 mm diameter was chosen. The height between the nozzle head and the printing bed was set to 0.2 mm. The samples were printed in the shape of cylinders (10 mm × 7 mm). All specimens were printed horizontally on the building platform with a raster angle of +45°/−45° in alternate layers and 100% infill density. Cylinders were printed with two different layer thicknesses, 0.1 and 0.2 mm, to evaluate the influence of the layer thickness on the final density and porosity of the printed samples. Table 3 presents an overview of the essential printing parameters employed for fabricating each sample, while Figure 2 depicts the spooled filaments and the printed samples.

### 2.3. Injection Moulding of Nd–Fe–B Bonded with Polyamide 12

Feeding pellets for injection moulding (IM) were made from filaments by crushing them into 5 to 10 mm pieces. The injection-moulded magnets were manufactured using Krauss Maffei KM50-190 at the manufacturing facilities at Kolektor KFH (Slovenia). The temperature range in the injection moulding unit ranged from 230 °C to 295 °C, with the pressure varying from 804 to 868 bar.

### 2.4. Characterization

Scanning electron microscopy coupled with energy dispersive X-ray analysis (SEM/EDX, JSM-IT300, JEOL) was used to analyse the shape and elemental composition of the as-received Nd–Fe–B powders. SEM images of the filaments were used to analyse the distribution of the filler particles in the polymer matrix. EDX was used to determine the elemental composition of the filaments and at the fracture of broken dog-bone-shaped tubes made from filament S90/10.

To evaluate the temperature stability and filler content in the filaments, thermogravimetric analysis (TGA) was performed using TGA/DSC 1 (Mettler-Toledo GmbH, Ljubljana, Slovenia). The filament samples were heated in air to 600 °C at a heating rate of 10 °C/min.

Differential scanning calorimetry (DSC) was used to evaluate the thermal stability of filaments and the influences of additives and fillers. The tested samples were heated and cooled in a thermal analyser apparatus (Mettler Toledo DSC1 STARe System). The first cooling run and second heating run were evaluated. The samples were heated from 25 °C to 480 °C at a rate of 10 °C/min in an air atmosphere to determine melting behaviour. The cooling cycle run between two heating cycles, from 200 °C to 30 °C at a rate of 10 °C/min in an air atmosphere, was carried out to determine cold crystallization. The degree of crystallinity, *X_c_* [%], was calculated based on the formula:

(1) $$X_{c}=\frac{\Delta H_{m}}{\left(1-w_{f}\right)*\Delta H_{0}}*100,$$

where Δ*H_m_* [J/g] is the melting enthalpy of filaments after the second heating, *w_f_* is the weight fraction of the filler [%], and Δ*H*_0_ [J/g] is the melting enthalpy [J/g] of 100% crystalline PA12 (245 J/g [12,25]). 

The rheological properties of the filaments were evaluated by measuring the melt flow index (MFI; LMI5000 Series, Dynisco). The filaments were cut into granules and 30 g of each batch was fed into an MFI capillary and preheated to 260 °C. The melt time was set to 120 s and a 5 kg load was applied.

The density of the printed cylinders was measured based on Archimedes’ principle using an analytical balance (XP205 by Mettler-Toledo GmbH). The measured densities were compared with the expected calculated densities. The expected density was calculated using the rule of mixtures, excluding void formation:

(2) $$\rho_{calculated}=\frac{\rho_{filler}*vol{\%}_{filler}+\rho_{add1}*vol{\%}_{add1}+\rho_{add2}*vol{\%}_{add2}+\rho_{add3}*vol{\%}_{add3}+\rho_{polymer}*vol{\%}_{polymer}}{100},$$

where labels *add*_1_, *add*_2_, and *add*_3_ refer to the following additives: coupling agent, internal lubricant, and external lubricant, respectively.

Porosity was evaluated using the following equation:

(3) $$Porosity\;\left(\%\right)=\frac{\rho_{calculated}-\rho_{measured}}{\rho_{calculated}}*100$$

FDM-printed cylinders were magnetized using an impulse magnetizer K-Series (MAGNET-PHYSIK) at a voltage of 2000 V to saturate the samples. After magnetization, the residual remanence (*B_r_*) and intrinsic coercivity (H_ci_) were measured using a permeameter (PERMAGRAPH^®^, MAGNET-PHYSIK). The measured residual remanence was compared with the theoretical value. The theoretical value of *B_r_* was calculated using the following formula:

(4) $$B_{r}{}_{theoretical}=\frac{vol{\%}_{filler}}{100}*B_{r}{}_{as-received\;powder}\;$$

The magnetic flux was measured using a Helmholtz coil (MS 75 with electronic Fluxmeter EF 14, MAGNET-PHYSIK) before and after exposure to different environmental tests.

### 2.5. Environmental Stability

Environmental stability of FDM-printed PBMs was studied using potential environmental stresses, i.e., accelerated hot aqueous immersion, dry heat, and cyclic temperature–humidity corrosion tests. The goal of these tests was to evaluate the effects of moisture, presence of aggressive ions, and temperature on the flux loss. Tests were performed with at least three samples.

Cylinders from batches B93/7 and S93/7, with a layer height of 0.1 mm, were immersed in deionized water at 85 °C for 1000 h to evaluate the influence of water absorption on flux loss. Cylinders from batches B90/10 and S90/10 with layer heights of 0.1 mm were immersed in corrosive water at a temperature of 95 °C for 1000 h. A solution of corrosive water was prepared according to the standard ASTM D1384 [26]. Sodium salts were dissolved in 1 L of deionized water. The exact amount of sodium salts can be found in Table 4.

To evaluate the effect of hot air, cylinders from batches B93/7 and S93/7 with a layer height of 0.1 mm, were exposed to dry air at 85 °C for 1000 h. Cylinders from batches B93/7 and S93/7 with a layer height of 0.1 mm were subjected to the Bulk Corrosion Test (BCT) according to ASTM A1071/A1071M-11 [27]. In the BCT test, the samples were exposed to pressurized steam to determine their resistance to degradation by the combined action of heat and water vapor. The samples were placed on top of glass, filled with demineralised water, and then placed in autoclaves at 120 °C for 96 h. The presence of water in the glass created 100% relative humidity at a pressure of 200 kPa. Four cylinders from batches B93/7 and S93/7 with a layer height of 0.2 mm were exposed to a Humidity Cyclic Test according to IEC-60068-2-38 [28]. The samples were kept in the environmental chamber for 10 cycles, with each cycle lasting 24 h. The first five cycles were a cold phase with a low temperature of 10 °C, and the five following cycles did not include a cold phase at a high temperature of +65 °C. The Humidity Cyclic Test is a type of environmental test used to evaluate the performance of materials and components under conditions involving changes in temperature and humidity. The test is designed to simulate real-world conditions and identify defects that may be caused by “breathing,” which is the movement of air or moisture in and out of a material or component. This can occur when the temperature on the surface of the material is lower than the dew point, resulting in condensation. As the temperature changes, the air inside the material expands and contracts, which can cause air or moisture to enter through the cracks or gaps. Over time, this can lead to water accumulation inside the material, potentially causing damage or failure.

The magnetic flux was measured using a Helmholtz coil before testing. The magnets were moved and cooled at the end of each test and the reversible flux loss was measured. The samples were saturated again to determine the irreversible flux loss and the flux was measured.

## 3. Results and Discussion

### 3.1. As-Received Magnetic Powder Characterization

**Figure 3:**
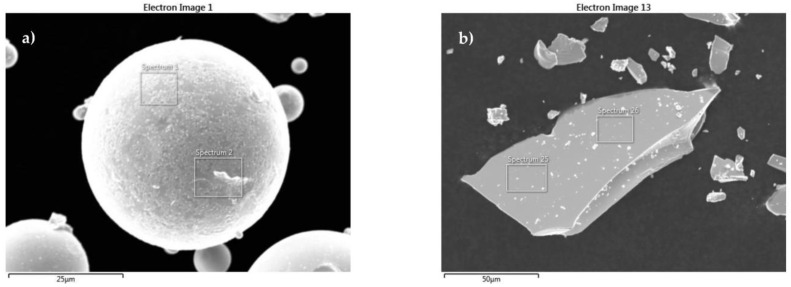
SEM micrographs of the as-received Mqp S particles (**a**) and Mqp B+ particles (**b**).

The morphology of the as-received Mqp S and Mqp B+ powders was examined using SEM. The SEM micrographs in Figure 3 show the effects of the different manufacturing methods. The melt-spun process produced irregularly shaped flake-like particles. The melt-spun process is followed by crushing; therefore, the Mqp B+ powder has a blocky morphology. Gas atomization produces spherical particles with smooth surfaces. For the Mqp B+ powder, a brittle nature can be observed. The SEM micrographs in Figure 3 show filler particles inside the polymer matrix in filaments. In filament B90/10, few Mqp S particles were observed because of the difficulty in thoroughly cleaning the extruder. In the subsequent subsection, the elemental composition of the as-received powders is discussed and compared with the elemental composition obtained for the magnetic portion of the filament.

### 3.2. Filaments Characterization

**Figure 4:**
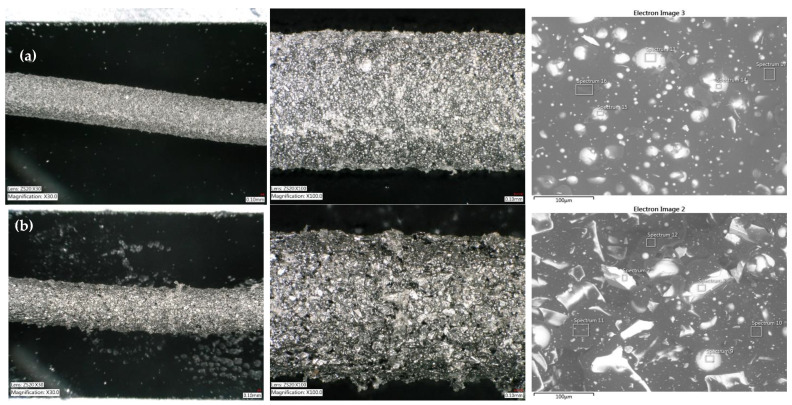
SEM micrographs of filament S90/10 (**a**) and filament B90/10 (**b**), showing Nd–Fe–B particles dispersed in the polymer matrix.

The surface features of filaments S90/10 and B90/10 are shown in Figure 4, indicating a contrast between the two. The former exhibits a relatively smoother surface texture owing to the finer magnetic particles used in its production. Conversely, the latter presents a more pronounced ‘shark skin’ appearance attributed to the use of coarser magnetic particles. Table 5 and Table 6 show the elemental compositions (wt.%) of the major elements detected at the surface of the as-received powders and filaments S90/10 and B90/10. The concentrations of Nd and Fe in the magnetic part of the filament were lower than those in the starting alloy, and the concentration of C was high. A high concentration of C could mean that the coupling agent TTS improved the adhesion between the filler and the polymer. Thus, a high C concentration originates from the polymer that encapsulates the Nd–Fe–B particles.

**Figure 5:**
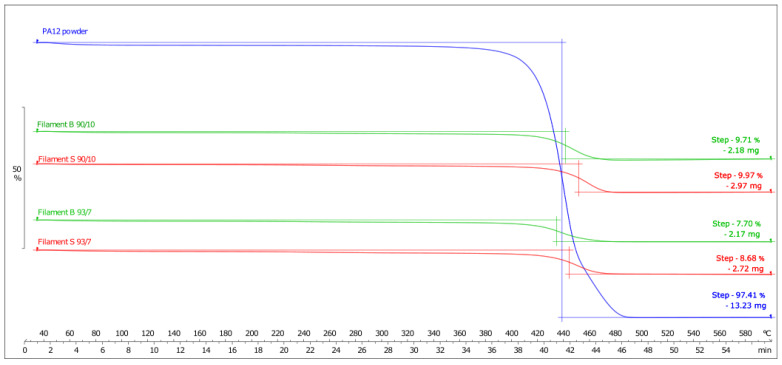
TGA curves after heating the filaments to 600 °C.

TGA analysis provided information on the mass reduction of the filament under a high-temperature condition. TGA analysis can be used to estimate the filler content in the filament, where the mass-loss percentage represents the degradation of the polymer. When filler loading was 93 wt.%, the expected mass loss would be 7%, and the same goes for filaments with 90 wt.%, for which the expected loss was 10%. However, Figure 5 shows that the loss is slightly higher and differs among the batches. The higher-than-expected mass loss can be attributed to the presence of additives, which also degrade to a certain extent. In addition, the higher mass loss suggests that the filler particles were partly degraded as a result of high temperatures.

The filament’s melting point was found to be approximately 177 °C through DSC analysis. This implies that FDM-printed magnets bonded with PA12 are capable of withstanding temperatures up to 170 °C. However, the operating temperature of printed magnets is contingent on their specific application and conditions, and it is restricted by the service temperature range of PA 12, which is −40 °C to 80 °C with short-term temperatures up to 110 °C. DSC data and the calculated degree of crystallinity are presented in Table 7, indicating that an increase in filler loading correlates with an increase in the degree of crystallinity and a decrease in melting temperatures. Furthermore, the data demonstrate a link between filler presence and the degree of crystallinity in FDM-printed specimens.

**Figure 6:**
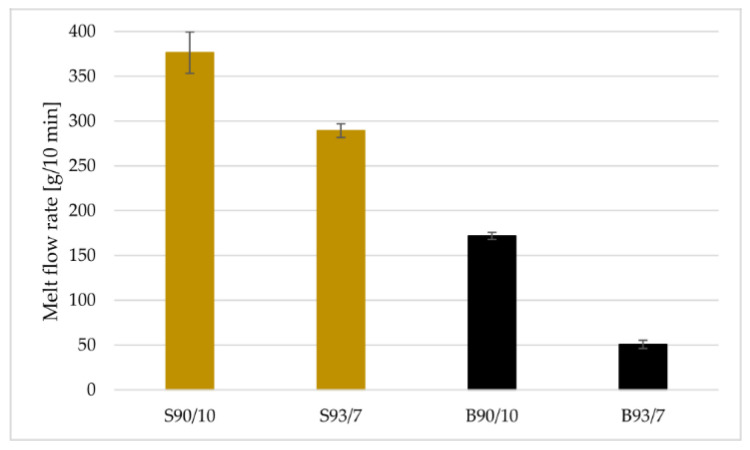
The melt flow rate [g/10 min] of Mqp S and Mqp B+ filaments with various filler loadings.

The MFR measurements, depicted in Figure 6, indicate that filaments with higher polymer content, such as S90/10 and B90/10, exhibit greater flowability than those with 7 wt.% of polymer binder. Hence, higher content of the thermoplastic binder leads to an increase in MFR values and improves fluidization. Additionally, the filament made with spherical particles had more than double the MFR values. These results also support the suitability of Mqp S powders for FDM printing, given their higher MFR values that reduce the risk of printer head clogging. Consequently, using Mqp S powder enables printing of larger and more complex-shaped samples.

### 3.3. Characterization of the FDM-Printed Polymer-Bonded Magnets

**Figure 7:**
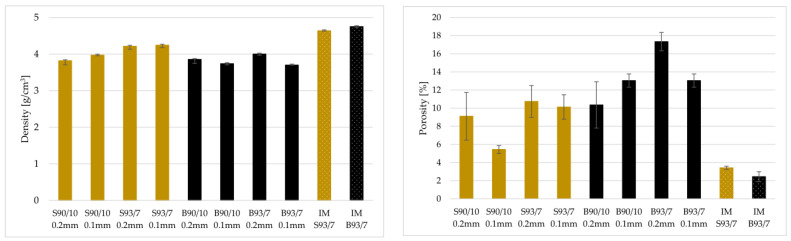
Bar charts of density and calculated porosity of FDM-printed magnets, with various filler loadings and printing heights, and injection-moulded magnets.

Poor density and porosity are common characteristics of materials produced by any additive manufacturing technology. Figure 7 presents bar graphs displaying the densities and estimated porosity of injection-moulded (IM) and FDM-printed magnets. FDM-printed magnets exhibited the lowest density, primarily due to poor adhesion between extruded layers and the lack of high pressure present in the IM process that brings the material together. Porosity was estimated from measured and calculated densities. Porosity in magnets can cause internal oxidation of the magnetic powder by air or moisture trapped inside the pores. Moreover, high porosity leads to low residual remanence due to low density. The measured density results reveal that samples printed with spherical powder possess higher density and porosity values than those printed with irregularly shaped powder. Moreover, S90/10 and S93/7 samples with a layer thickness of 0.1 mm demonstrate higher density and lower porosity values compared to those with a layer thickness of 0.2 mm. These findings indicate that the shape and size of the powder particles, as well as the layer thickness, play a crucial role in the final density and porosity of FDM-printed samples.

**Figure 8:**
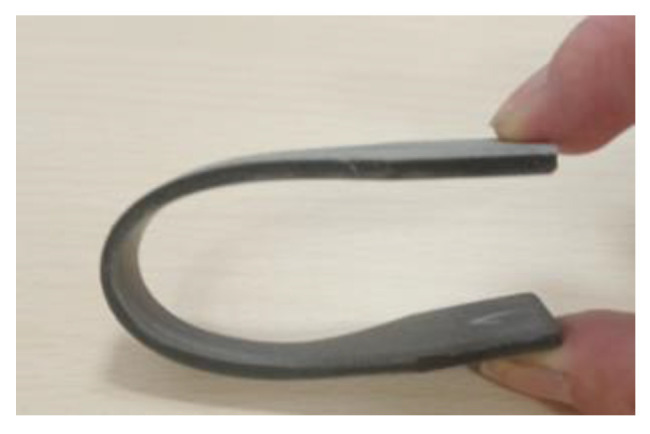
Dog-bone-shaped specimen, FDM-printed from filament S90/10, fully bended without breaking.

A dog-bone-shaped specimen (type 1 B) was printed using filament S90/10 by FDM printing, in accordance with ISO 527-2 [24]. This type of specimen is commonly used in mechanical testing, specifically for flexural and tensile testing, in order to evaluate the strength and behaviour of the material under load. The flexibility of the material made it challenging to conduct mechanical tests, as the specimens did not break under an applied load of 10 kN. A flexible specimen is depicted in Figure 8.

**Figure 9:**
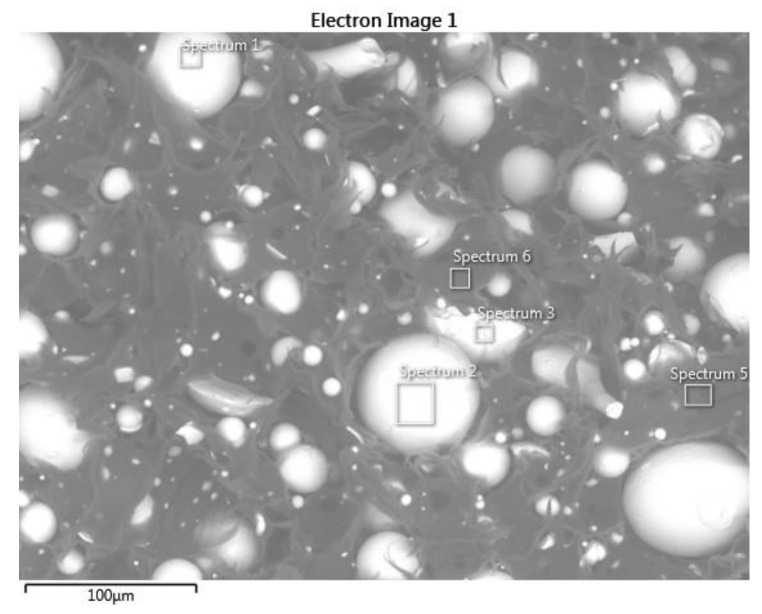
SEM image of surface fracture of dog-bone-shaped specimen, FDM-printed from filament S90/10. Nd–Fe–B were pulled out of the polymer matrix.

A tensile test with the same specimens was repeated until the specimen broke. After the tensile test, the surface fracture of the specimen was examined using SEM/EDX. A SEM image of the specimen is shown in Figure 9. The carbon content values on powder particles, as determined through EDX analysis in Table 8, are consistent with the results obtained from the EDX analysis of the S90/10 filament from Table 5.

The magnetic properties of the samples were evaluated and the results for at least three samples are summarized in Table 9. The expected lower Br values of FDM-printed samples due to their low density and high porosity were observed. However, considering the constraints of FDM printing technologies (lack of high pressure), extremely high Br values were obtained for the FDM-printed samples. The Br values of the FDM-printed samples were above 90% of the expected theoretical remanence for a filler loading of 90 wt.%. FDM magnets made from Mqp S achieved a Br value of 98% for 93 wt.% filler loading, while those made from Mqp B had 87%. The intrinsic properties of the material, such as coercivity (Hci), depend on the composition of the starting material and are not affected by the sample density. The Hci values of the as-received powder, as stated in MDS, are 716–836 kA/m for the melt-spun powder and 670–750 kA/m for the atomized gas. The Hci values of the FDM-printed samples were consistent with those of the as-received powder and the IM samples, indicating that no degradation of the magnetic powder occurred during filament manufacturing and FDM printing.

Total flux loss can be categorized into reversible loss, recoverable irreversible loss, and structural loss. Reversible flux loss occurs as a function of temperature and can be undone by cooling the magnet. Irreversible changes are those that do not return to their original value after the disturbing influence is removed. These can be further divided into those that can be restored by remagnetisation at room temperature, recoverable irreversible loss, and those that cannot, as a result of structural or metallurgical changes, often called aging loss [29].

**Figure 10:**
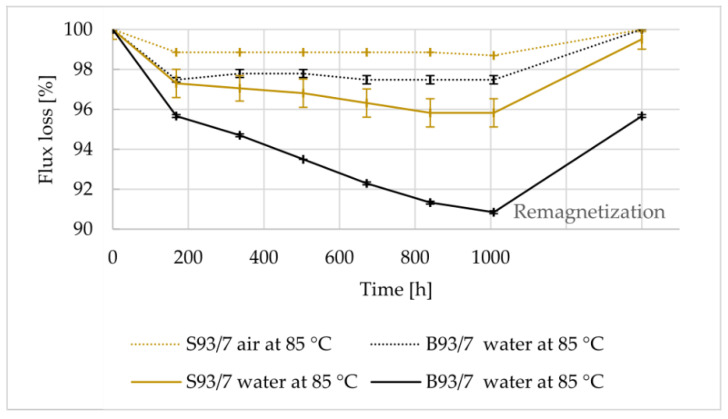
Flux loss of FDM-printed samples S93/7 and B93/7 vs. time exposure to hot water and air at 85°C, and after final remagnetization.

**Figure 11:**
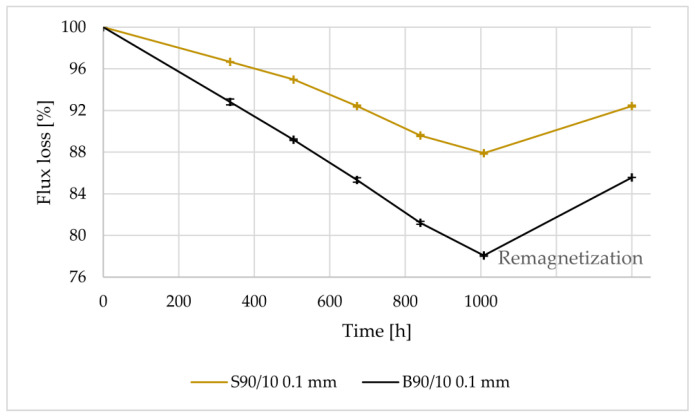
Flux loss of FDM-printed samples S90/10 and B90/10 vs. time exposure to corrosive water at 95°C for 1000 h and after final remagnetization.

**Figure 12:**
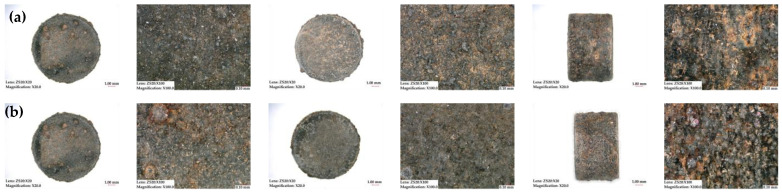
Surface corrosion of samples S90/10 (**a**) and B90/10 (**b**): top, bottom, and side after immersion in corrosive water at 95 °C for 1000 h.

**Figure 13:**
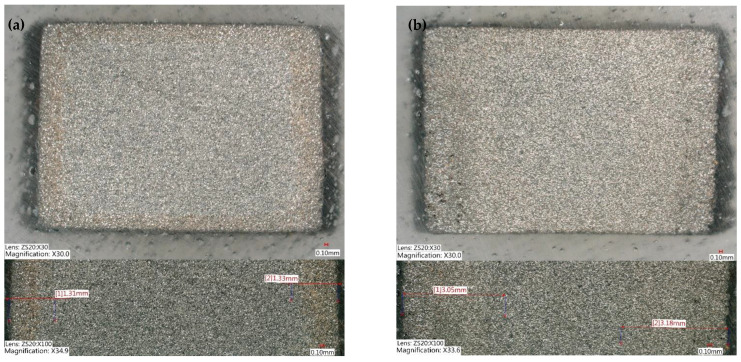
Surface corrosion, cross-section of magnets S90/10 (**a**) and B90/10 (**b**) showing progress of corrosion after immersion in corrosive water at 95 °C for 1000 h.

The reversible and irreversible flux losses were evaluated after exposing the magnets to different scenarios. It is likely that the magnets made from the gas-atomized powder experienced a lower flux loss because they had a higher density and lower porosity. This implies that if a magnet is more compact and has fewer gaps or voids, it has less possible routes for water or air entrapment. A higher density and lower porosity can lead to improved magnetic performance, resulting in lower flux loss. The rule of thumb in industry is that magnets should not have flux loss higher than 5% over 1000 h of testing [9,30]. Table 10 provides a summary of the environmental tests performed to evaluate the stability of the magnets, as well as the final irreversible flux loss observed after testing. The results indicate that irreversible flux loss was less than 5% in all tests except for the test where FDM magnets were immersed in corrosive water. It is worth noting that this particular test is considered highly aggressive and it is uncertain whether even IM magnets would have a flux loss of less than 5%. The flux loss of magnets produced using Mqp S powder was lower than those made from Mqp B+ when immersed in pure water, as shown in Figure 10. This difference may be attributed to the better bonding between magnet and polymer particles in the former. However, in corrosive water solution with aggressive ions at high temperatures, the presence of aggressive ions accelerates surface rusting and flux loss, as depicted in Figure 11 and Figure 12, for both S90/10 and B90/10 magnets. Figure 13 shows a cross-section of the most corroded sample after the corrosive water test, which demonstrates the progression of corrosion in the FDM samples. Sample B90/10 had a significantly higher depth of corrosion, nearly double than that of S90/10, and exhibited almost double the flux loss after the same test.

## 4. Conclusions

Fused deposition modelling (FDM) technology can be utilized to produce Nd–Fe–B magnets bonded with PA12 in a cost-effective manner. However, FDM-printed magnets exhibit lower magnetic properties than their injection-moulded counterparts, possibly due to their lower density and higher porosity. Nonetheless, FDM-printed magnets can achieve high magnetic properties by utilizing high loading factors. For instance, FDM magnets made from Mqp S had a Br value of 98% of the expected theoretical remanence for 93 wt.% filler loading, while those made from Mqp B had 87%. The extrusion process used in filament making and FDM printing did not affect the coercivity of the samples. Furthermore, FDM-printed magnets exhibited an irreversible flux loss of less than 5% or zero when exposed to hot water, air, or pressurized steam. Thus, FDM-printed magnets are suitable for applications involving such conditions. By using a coupling agent, the adhesion between the magnetic filler and polymer binder was improved, as confirmed by SEM/EDX analysis. Overall, although FDM printing is a low-budget option for producing Nd–Fe–B magnets, it can generate magnets with comparable magnetic performance and corrosion resistance to those produced via injection moulding.

## Figures and Tables

**Table 1:** Magnetic properties of the as-received Nd–Fe–B powders (source material datasheet: https://mqitechnology.com/products/bonded-neo-powder).

Magnetic Powder	Residual Induction, Br [mT]	Intrinsic Coercivity, Hci [kA/m]	Energy Product, (BH)_max_ [kJ/m^3^]
Mqp S	730–760	670–750	80–92
Mqp B+	895–915	716–836	126–134

**Table 2:** Composition of the prepared filaments.

Sample	Nd–Fe–B Powder	Nd–Fe–B		PA12	
		[wt.%]	[vol.%] *	[wt.%]	[vol.%] *
S90/10	Spherical	90	57.1	10	41.6
S93/7	Spherical	93	66.1	7	32.6
B90/10	Irregular shape	90	56.5	10	65.5
B93/7	Irregular shape	93	42.2	7	33.2

* Including added additives and excluding estimated void of 1.3%.

**Table 3:** Overview of printing parameters.

Printing Parameter	Value [Unit]
Nozzle temperature	250 °C
Printing speed	10 mm/s
Platform temperature	70 °C
Nozzle head diameter	0.8 mm
Raster angle	+45°/−45°
Infill density	100%
Layer thicknesses	0.1 mm
	0.2 mm

**Table 4:** Chemical composition of the corrosive water solution.

Compound	Concentration [mg/L]
Na_2_SO_4_	148
NaCl	165
NaHCO_₃_	138

**Table 5:** Elemental composition (wt.%) of the as-received Mqp S powder and filament S90/10.

Spectrum Label	As-Received Mqp S Powder		Mqp S Powder in Filament S90/10			PA12 Matrix in Filament S90/10	
	Sp. 1	Sp. 2	Sp. 13	Sp. 14	Sp. 15	Sp. 16	Sp. 17
Fe	73.7	73.7	27.1	26.1	26.2	8.4	6.9
Nd	17.4	17.2	7.0	6.9	6.5	2.3	1.9
C			56.7	57.7	58.1	75.8	77.5
O			6.4	7.0	6.6	12.3	12.7
Ti	2.3	2.1	0.9	0.9	0.9	0.5	0.4
Zr	6.4	3.9	2.0	1.5	1.7	0.7	0.7
Co	3.0	3.2					
Total	100.0	100.0	100.0	100.0	100.0	100.0	100.0

**Table 6:** Elemental composition (wt.%) of the as-received Mqp B+ powder and filament B90/10.

Spectrum Label	As-Received Mqp B+ Powder		Mqp B+ Powder in Filament B90/10			PA12 Matrix in Filament B90/10		
	Sp. 25	Sp. 26	Sp. 7	Sp. 8	Sp. 9	Sp. 10	Sp. 11	Sp. 12
Fe	66.3	66.0	27.4	27.0	30.0	8.6	9.7	7.9
Nd	28.0	28.3	11.9	11.8	8.4	3.5	4.0	3.3
C			51.9	52.7	53.3	75.7	74.6	75.4
O			5.9	6.2	5.5	11.3	10.8	12.6
Co	5.7	5.7	2.2	2.2	1.3	0.6	0.6	0.6
Ni			0.4					
Zr			0.2	0.2	1.6	0.4	0.3	0.3
Total	100.0	100.0	100.0	100.0	100.0	100.0	100.0	100.0

**Table 7:** DSC analysis on the filament samples. The table includes data on the melting temperature after first and second heating, T_m1_ and T_m2_, respectively; enthalpy after first and second heating, Δ*H*_*m*1_ and Δ*H*_*m*2_, respectively; cold crystallization temperature, T_c_, and enthalpy, *H_c_*, after the cooling cycle; and the calculated degree of crystallinity, *X_c_*.

	T_m1_ (°C)	Δ*H*_*m*1_ (J/g)	T_c_ (°C)	Δ*H*_*c*_ (J/g)	T_m2_ (°C)	Δ*H*_*m*2_ (J/g)	*X_c_* (%)
S90/10	177.7	6.1	149.7	6.0	176.1	5.3	21.6
S93/7	176.2	5.1	149.9	4.5	174.6	4.1	23.9
B90/10	176.8	5.5	149.4	5.9	175.9	5.3	21.7
B93/7	176.2	4.2	147.8	4.2	175.4	3.8	22.3

**Table 8:** Elemental composition (wt.%) of the surface fracture of dog bone specimen made from filament S90/10.

Spectrum Label	Mqp S Powder in Test Tube			PA12 Matrix in Test Tube	
	Sp. 1	Sp. 2	Sp. 3	Sp. 5	Sp. 6
Fe	38.4	39.8	38.5	17.4	19.1
Nd	10.4	10.4	10.0	4.7	5.2
C	43.2	42.5	44.7	68.0	66.8
O	4.3	3.6	3.5	8.1	7.2
Ti	1.2	1.3	1.2	0.8	0.7
Zr	2.6	2.4	2.1	1.1	1.1
Total	100.0	100.0	100.0	100.0	100.0

**Table 9:** Measured magnetic properties of FDM-printed and injection-moulded (IM) samples.

	BH_max_ [kJ/m^3^]	H_ci_ [kA/m]	B_r measured_ [mT]	B_r theoretical_ [mT]
S90/10 0.2 mm	18.7 (±0.9)	719.2 (±1.2)	347.8 (±0.4)	375.1
S90/10 0.1 mm	21.7 (±0.1)	721.8 (±1.2)	367.3 (±0.4)	375.1
S93/7 0.2 mm	28.9 (±1.1)	721.5 (±1.3)	422.2 (±7.9)	434.2
S93/7 0.1 mm	29.4 (±1.5)	721.3 (±2.6)	426 (±11.0)	434.2
B90/10 0.2 mm	27.3 (±0.4)	700.6 (±0.8)	410.8 (±3.4)	449.5
B90/10 0.1 mm	28.7 (±0.4)	712.2 (±1.3)	409.8 (±2.5)	449.5
B93/7 0.2 mm	35.5 (±1.0)	713.2 (±0.9)	456.5 (±6.8)	521.4
B93/7 0.1 mm	35 (±0.3)	705.8 (±1.0)	454.2 (±1.7)	521.4
IM S93/7	32 (±0.1)	710.8 (±1.1)	449 (±0.7)	434.2
IM B93/7	46.3 (±0.6)	697.7 (±1.0)	528.8 (±3.3)	521.4

**Table 10:** Overview of environmental testing and flux loss due to aging.

Test Name	Test Temperature/Duration	Sample Name	Reversible Flux Loss [%]	Irreversible Flux Loss [%]	Rusting
Immersion in water	85 °C/1000 h	S93/7 0.1 mm	3.4	0.5	Low
		B93/7 0.1 mm	9.2	4.3	Low
Exposure to hot air	85 °C/1000 h	S93/7 0.1 mm	1.3	0	No evidence
		B93/7 0.1 mm	2.5	0	No evidence
Immersion in corrosive water	95 °C/1000 h	S90/10 0.1 mm	12.1	7.6	Severe
		B90/10 0.1 mm	21.9	14.4	Severe
Bulk corrosion test	120 °C/96 h	S93/7 0.2 mm	2.9	0	Low
		B93/7 0.2 mm	4.8	0	Low
Cyclic temperature/humidity test	−10°C/65°C/240 h	S93/7 0.2 mm	0.6	0	No evidence
		B93/7 0.2 mm	0.8	0	No evidence

## Data Availability

Not applicable.

## References

[1] Brown D., Ma B.M., Chen Z. (2002). Developments in the Processing and Properties of NdFeb-Type Permanent Magnets. J. Magn. Magn. Mater..

[2] Liu J., Walmer M., Liu Y., Sellmyer D.J., Shindo D. (2006). Process and Magnetic Properties of Rare-Earth Bonded Magnets. Handbook of Advanced Magnetic Materials.

[3] (2015). Standard Terminology for Additive Manufacturing—General Principles—Terminology.

[4] Moradi M., Aminzadeh A., Rahmatabadi D., Hakimi A. (2021). Experimental Investigation on Mechanical Characterization of 3D Printed PLA Produced by Fused Deposition Modeling (FDM). Mater. Res. Express.

[5] Rahmatabadi D., Ghasemi I., Baniassadi M., Abrinia K., Baghani M. (2022). 3D Printing of PLA-TPU with Different Component Ratios: Fracture Toughness, Mechanical Properties, and Morphology. J. Mater. Res. Technol..

[6] Moradi M., Aminzadeh A., Rahmatabadi D., Rasouli S.A. (2021). Statistical and Experimental Analysis of Process Parameters of 3D Nylon Printed Parts by Fused Deposition Modeling: Response Surface Modeling and Optimization. J. Mater. Eng. Perform..

[7] Ngo T.D., Kashani A., Imbalzano G., Nguyen K.T.Q., Hui D. (2018). Additive Manufacturing (3D Printing): A Review of Materials, Methods, Applications and Challenges. Compos. B Eng..

[8] Huber C., Abert C., Bruckner F., Groenefeld M., Muthsam O., Schuschnigg S., Sirak K., Thanhoffer R., Teliban I., Vogler C. (2016). 3D Print of Polymer Bonded Rare-Earth Magnets, and 3D Magnetic Field Scanning with an End-User 3D Printer. Appl. Phys. Lett..

[9] Li L., Jones K., Sales B., Pries J.L., Nlebedim I.C., Jin K., Bei H., Post B.K., Kesler M.S., Rios O. (2018). Fabrication of Highly Dense Isotropic Nd-Fe-B Nylon Bonded Magnets via Extrusion-Based Additive Manufacturing. Addit. Manuf..

[10] Paranthaman M.P., Yildirim V., Lamichhane T.N., Begley B.A., Post B.K., Hassen A.A., Sales B.C., Gandha K., Nlebedim I.C. (2020). Additive Manufacturing of Isotropic NdFeB PPS Bonded Permanent Magnets. Materials.

[11] Palmero E.M., Casaleiz D., Jiménez N.A., Rial J., de Vicente J., Nieto A., Altimira R., Bollero A. (2019). Magnetic-Polymer Composites for Bonding and 3D Printing of Permanent Magnets. IEEE Trans. Magn..

[12] Slapnik J., Pulko I., Rudolf R., Anžel I., Brunčko M. (2021). Fused Filament Fabrication of Nd-Fe-B Bonded Magnets: Comparison of PA12 and TPU Matrices. Addit. Manuf..

[13] Pigliaru L., Rinaldi M., Ciccacci L., Norman A., Rohr T., Ghidini T., Nanni F. (2020). 3D Printing of High Performance Polymer-Bonded PEEK-NdFeB Magnetic Composite Materials. Funct. Compos. Mater..

[14] von Petersdorff-Campen K., Hauswirth Y., Carpenter J., Hagmann A., Boës S., Schmid Daners M., Penner D., Meboldt M. (2018). 3D Printing of Functional Assemblies with Integrated Polymer-Bonded Magnets Demonstrated with a Prototype of a Rotary Blood Pump. Appl. Sci..

[15] Yang F., Zhang X., Guo Z., Ye S., Sui Y., Volinsky A.A. (2019). 3D Printing of NdFeB Bonded Magnets with SrFe12O19 Addition. J. Alloys Compd..

[16] Shen A., Peng X., Bailey C.P., Dardona S., Ma A.W.K. (2019). 3D Printing of Polymer-Bonded Magnets from Highly Concentrated, Plate-like Particle Suspensions. Mater. Des..

[17] Hsiang Loh G., Pei E., Gonzalez-Gutierrez J., Monzón M. (2020). An Overview of Material Extrusion Troubleshooting. Appl. Sci..

[18] Sojer D., Skulj I., Kobe S., Kovač J., McGuiness P.J. (2013). Protection of Nd2Fe14B-Based Melt-Spun Ribbons Using Nanoscale Sol–Gel Derived Films of SiO2 and Al2O3. Surf. Coat. Technol..

[19] Liu W., Yang Y., Meng Y., Wu J. (2003). The Effects of Surface Modification on the Properties of Bonded NdFeB Magnets. Mater. Trans..

[20] Gardocki A. Investigation of the Thermo-Oxidative Degradation of Plastic Bonded Rare-Earth-Magnets during the Injection Molding Process. Proceedings of the 2011 1st International Electric Drives Production Conference.

[21] Xiao J., Otaigbe J. (2000). Polymer-Bonded Magnets: III. Effect of Surface Modification and Particle Size on the Improved Oxidation and Corrosion Resistance of Magnetic Rare Earth Fillers. J. Alloys Compd..

[22] Ma B.M., Herchenroeder J.W., Smith B., Suda M., Brown D., Chen Z. (2002). Recent Development in Bonded NdFeB Magnets. J. Magn. Magn. Mater..

[23] Fan Y.-L., Hwang K.-S. (2007). Properties of Metal Injection Molded Products Using Titanate-Containing Binders. Mater. Trans..

[24] Nor S.S.M., Rahman M.M., Tarlochan F., Shahida B., Ariffin A.K. (2008). The Effect of Lubrication in Reducing Net Friction in Warm Powder Compaction Process. J. Mater. Process. Technol..

[25] Xenopoulos A., Wunderlich B. (1990). Thermodynamic Properties of Liquid and Semicrystalline Linear Aliphatic Polyamides. J. Polym. Sci. B Polym. Phys..

[26] (2019). Standard Test Method for Corrosion Test for Engine Coolants in Glassware.

[27] (2015). Standard Test Method for Evaluating Hygrothermal Corrosion Resistance of Permanent Magnet Alloys.

[28] Environmental Testing—Part 2: Tests-Test Z/AD: Composite Temperature/Humidity Cyclic Test (IEC 60068-2-38:2009). https://standards.iteh.ai/catalog/standards/clc/ffb7b494-b202-4ffb-90e2-c0d8555db92e/en-iec-60068-2-38-2021.

[29] Leonowicz M., Kaszuwara W., Wojciechowski S., Davies H.A. (1996). Irreversible Losses of Magnetisation in Fe–Nd–B Type Magnets. J. Magn. Magn. Mater..

[30] Noguchi K., Mishima C., Yamazaki M., Matsuoka H., Mitarai H., Honkura Y. Development of Dy-Free NdFeB Anisotropic Bonded Magnet (New MAGFINE). Proceedings of the 2011 1st International Electric Drives Production Conference.

